# A description of COVID-19 related delusional content in admissions to an acute psychiatric unit

**DOI:** 10.4102/sajpsychiatry.v30i0.2275

**Published:** 2024-07-16

**Authors:** Marc D. Stopford, Alexandra Maisto, Wendy Friedlander

**Affiliations:** 1 Department of Psychiatry, Faculty of Health Sciences, University of the Witwatersrand, Johannesburg, South Africa

**Keywords:** COVID-19, delusional content, pathoplasticity, coronavirus 2019, COVID-19 psychiatry, delusional plasticity, external factors of delusions, COVID delusions, COVID-19 delusions

## Abstract

**Background:**

The COVID-19 pandemic had a profound global impact, affecting individuals, including those with mental illness, through early and widespread information dissemination. Although the neurobiological basis of delusions remains unclear, external stimuli and historical events are known to influence them. The pandemic provided a unique opportunity to explore this phenomenon.

**Aim:**

To determine the prevalence of COVID-19-related delusional content, among individuals presenting for treatment of psychosis during the peak of the COVID-19 pandemic and investigate associated clinical and demographic factors.

**Setting:**

Chris Hani Baragwanath Academic Hospital in-patient psychiatry department.

**Methods:**

Data were extracted retrospectively from adult psychiatric admissions spanning April to September 2020 on patients whose presenting complaints included delusions. Demographic factors, symptoms, psychiatric, medical and substance use history, and a documented Diagnostic and Statistical Manual of Mental Disorders, Fifth Edition (DSM-5) diagnosis by the attending psychiatrist were collected.

**Results:**

The prevalence of COVID-19-related delusional content was 25.5%. Significant demographic association was observed with education level of Grade 12 and above (*p* = 0.000338). The odds of a diagnosis of schizophrenia and related disorders were 2.72 times greater than mood and psychotic disorder due to another medical condition in those with COVID- 19-related delusional content (OR 2.19, 95% CI: [1.4-3.4]).

**Conclusion:**

The presence of COVID-19-related delusional content in patients admitted to hospital with psychosis provides further evidence of the role of external stimuli in the formation of delusions.

**Contribution:**

This study underscores the influence of socio-cultural factors on delusions and advocates for interventions and expanded research to address mental health outcomes.

## Introduction

The coronavirus disease 2019 (COVID-19) pandemic, which commenced in the latter part of 2019 (December), had an unprecedented influence on the global population in medical, psychological and socio-economic terms. The impact on mental health extended beyond those directly affected by the virus to encompass individuals who were previously in good mental health, as well as those with pre-existing mental illnesses. In an evaluation of available meta-analyses and systematic reviews conducted over the pandemic period, the World Health Organization (WHO) documented a 25% surge in common mental disorders such as depression and anxiety.^1^ There is a dearth of literature on the prevalence of psychosis and/or psychotic disorders during the same period; however, available evidence suggests an increase in the risk of first-time psychotic symptoms, relapses in previously stable patients and the worsening of symptoms in individuals with psychotic disorders.^2,3^ The hypothesised triggers were multifactorial and included social isolation, increasing financial burden, the fear of death, infection and the loss of loved ones.^4,5^

In response to the rapid and devastating spread of COVID-19, governments worldwide implemented regulations to curb travel, advised social isolation and enforced varying degrees of economic restrictions to protect their healthcare systems. These measures disproportionately impacted marginalised communities, including the unemployed, low-income earners, women, migrants and disabled populations.^5^ Developing countries, in particular, faced the need for stricter lockdowns because of limited capacity for social relief interventions and already strained healthcare infrastructure.^6,7,8^ South Africa was similarly affected. As of February 2024, the country recorded 4 092 483 positive cases and 102 595 deaths.^9^

Individuals with psychosis have been identified as one of the most vulnerable to the social determinants of health.^6^ For example, individuals with schizophrenia often face challenges such as compromised physical well-being, greater socio-economic disadvantages, social disconnection and pervasive stigma and discrimination.^7^ Movement restrictions and the fear of infection may impede help-seeking behaviours among this population, contribute to treatment non-adherence and result in inadequate follow-up care, thereby elevating the risk of relapse.^7^ The imposition of social isolation further exacerbated the experience of alienation potentially intensifying referential thinking and suspiciousness associated with psychosis.^8^

Psychosis refers to an aberration in thoughts, beliefs and perception that can lead to a detachment from reality.^10^ It encompasses a range of symptoms including delusions, hallucinations, disorganised speech and disorganised behaviour. Psychosis can manifest as a symptom of various mental disorders such as schizophrenia and mood disorders, as well as being linked to specific medical conditions and substance use.^10^

A delusion is characterised as a persistent, false belief that contradicts the cultural or religious norms of an individual.^10^ Delusions are steadfastly maintained despite overwhelming evidence to the contrary.^11^ Currently, there is no specific explanation regarding the neurobiological abnormalities that underlie delusions, rather they are believed to arise from a complex interplay of biological, psychological and social factors.^12,13^

Delusions exist within subjective and interpersonal dimensions and have infinite variations in presentation. Nevertheless, there appears to be a shared experience of delusional themes across different contexts and disorders. The international literature consistently recognises delusional themes such as persecution, grandiosity, guilt, religion, hypochondria, jealousy and love across multiple settings.^14^

Kurt Schneider theorised that the development of delusions occurred in three phases.^15^ The first phase is the initiation or basal irritation phase. The second phase is the externalisation phase. Lastly, the third phase is the concretisation phase, during which fully formed delusional perceptions and experiences of being influenced occur and become concrete for the patient.^14^ It is within the concretisation phase that socio-cultural, technological innovations and historical or political events may shape an individual’s delusions in the context of psychosis.^14^ These events and current affairs help provide answers to questions like ‘by whom’ and ‘in what way’. The fundamental psychotic symptoms integrate with ideas of personal significance or the individual experiences of the patient.^14^

It has additionally been demonstrated and widely accepted that delusional themes exhibit long-term stability.^14^ However, the content of these themes can vary in different eras, political climates and socio-cultural settings. Fundamental beliefs and socio-cultural factors play a role in determining the interpretive framework through which psychotic experiences are understood and given meaning.^16^ Studies conducted in various populations, including in the United States of America (US), China, Korea and Slovenia, have shown that delusional content reflects socio-cultural, technological, historical and political changes of a particular era.^11,13,14,17,18,19,20,21,22^

With the onset of the COVID-19 pandemic, mental health clinicians reported anecdotal evidence of increased paranoia among individuals, particularly regarding contamination from people in their immediate vicinity.^23^ This was followed by several case reports from Germany, Spain, Iran and India describing patients with delusional beliefs specifically associated with COVID-19 and the pandemic, either as known cases or first presentations.^23,24,25,26,27^ Notably, these delusions often centred around China and were influenced by information and misinformation circulated through social media. Some of these reports even preceded the arrival of the virus in certain regions by several days.^23^

The rapid propagation of publicity surrounding COVID-19 is a crucial factor to consider when exploring the timeline for the emergence of delusional beliefs. Few global events have captured the attention of the world’s population as swiftly as the COVID-19 pandemic. While previous outbreaks such as the 2014 Ebola crisis, the 2015 Zika outbreak, the 2009 H1N1 pandemic and the 2012 Middle Eastern Respiratory Syndrome (MERS-CoV) received significant media coverage, the speed and magnitude of information dissemination during this pandemic surpassed them all.^22^

The Internet and social media have been an increasingly utilised source of public health information dissemination worldwide. Their utilisation may have allowed a feeling of connectedness in the context of international lockdowns and social isolation. However, the increasing reliance on unregulated social media content has also created the opportunity for the spread of misinformation and fake news. The infodemic – a term first coined by David Rothkopf during the severe acute respiratory syndrome (SARS) epidemic in 2003 – describes the spread of misinformation and fake news during a health crisis. Misinformation is false or inaccurately presented information, whether intentional or not, often spread without the intent to harm. Fake news refers specifically to fabricated or fraudulent new stories particularly prevalent on social media, which distort or manipulate facts and can spread rapidly. The impact of fake news extends to perpetuating conspiracy theories, like attributing COVID-19 to a Chinese biological weapon or suggesting unproven treatments such a lemon with water or certain drugs. This dissemination of misleading health information leads to real-world consequences.^28^ The overwhelming often contradicting information results in fear and anxiety and makes it difficult for individuals to evaluate the veracity of the information.^29^ The consequences, subtle and life threatening, have been well documented. For instance, the promotion of chlorine dioxide products as a cure in the early stages of the pandemic led to a surge in accidental poisonings.^30^ Hydroxychloroquine overdoses were reported on, as well as drug shortages and panic buying.^28^ Similarly, the spread of misinformation suggesting that COVID-19 vaccines were a means to implant trackable microchips impacted uptake of this preventative health intervention.^29^

The term ‘pathoplasticity’ coined by Zutt in 1967 underscores the idea that cultural factors can shape the expression and presentation of mental disorders, including psychosis. While it is widely acknowledged that cultural patterns can influence psychotic features, quantifying the extent of this influence remains a challenge. Cultural influences extend beyond socialisation, religion, symbols and values to encompass environmental factors such as major events and climate. These diverse cultural elements can significantly impact the manifestation of psychotic symptoms.^31^ Studies and case reports have demonstrated the pathoplasticity of delusions, which serves as a crucial component in comprehending psychosis.^14^ The COVID-19 pandemic, with its profound global impact and far-reaching consequences, presents a unique opportunity to investigate how the external world can influence the manifestation of psychotic symptoms.

Existing research has primarily delved into the repercussions of the COVID-19 pandemic on both pre-existing and emerging mental health ailments. Noteworthy attention has been directed towards understanding manifestations of anxiety, depression and their effects on severe mental illnesses secondary to the COVID-19 pandemic.^4,5,7,32,33,34,35^ Since the early stages of the pandemic, numerous case studies documenting delusional episodes linked to COVID-19 content have surfaced and persist in scholarly discourse. Moreover, investigations have honed in on exploring themes of conspiracy ideation and alterations in thought content among affected individuals.^23,24,25,26,27^

South Africa has a substantial burden of mental illness with individuals facing numerous socio-economic risk factors, including a high prevalence of unemployment, poverty and comorbidity with human immunodeficiency virus (HIV).^35^ Furthermore, there exists a notable treatment gap, with mental health concerns often falling short of being prioritised.^36^ As the world adjusts to life in the aftermath of the COVID-19 pandemic, it is important to anticipate the potential increase in the demand for mental health services because of longer-term neuropsychiatric effects and psychological consequences of COVID-19. Understanding how COVID-19 impacted the presentation of psychosis can help healthcare systems better respond to similar situations in the future.

### Aim and objectives

This study aimed to describe the prevalence of COVID-19-related delusional content, among individuals presenting with psychosis during the peak of the COVID-19 pandemic and explore whether there is an association between their demographic and clinical factors and the COVID-19-related delusional content.

The objectives of this study were:

- To describe the prevalence and type of COVID-19-related delusional content among acute psychiatric admissions to a tertiary-level general hospital in an urban area over 6-month period (01 April 2020–30 September 2020)
- To describe the relationship between certain socio-demographic features and the presence of COVID-19-related delusional content.
- To describe the relationship between COVID-19-related delusional content and the patient’s diagnosis.

## Research methods and design

### Study design

This is a retrospective, quantitative, record review.

### Study setting

The study was conducted at Chris Hani Baragwanath Academic Hospital (CHBAH) in-patient psychiatry department. Chris Hani Baragwanath Academic Hospital is a tertiary-level general hospital located in Soweto, Johannesburg, South Africa. The hospital serves a large, urban population and consequently, the psychiatric unit manages significant numbers of acute and chronic patients, both previously treated and index presentations.

### Study population and sampling strategy

The hospital records of all psychiatric admissions for the 6-month period of 01 April 2020–30 September 2020 were reviewed from the in-patient register in the psychiatric department. The principal investigator screened files for eligibility criteria. Inclusion criteria comprised all adult patients admitted to any psychiatric ward at CHBAH between 01 April 2020 and 30 September 2020 who exhibited psychosis with delusions The exclusion criteria encompassed all patients under the age of 18, with absent delusions.

### Data collection

The reviewed inpatient records included all notes from the time of admission to discharge. The patients had received a structured assessment on admission which includes their demographic factors, presenting symptoms, psychiatric, medical and substance use history and a documented Diagnostic and Statistical Manual of Mental Disorders, Fifth Edition (DSM-5) diagnosis by the attending psychiatrist on the discharge report, as is routine for all admissions at CHBAH. Patients were interviewed by trainee psychiatrists and medical officers in psychiatry, and the final diagnosis was reviewed by the supervising psychiatrist. Sociodemographic (sex; age; the highest level of education; marital status; employment status) and clinical data (presenting symptoms, delusion classification and diagnostic information, past psychiatric history and substance use) were collected and entered onto a data sheet linked to an Excel spreadsheet.

### Data analysis

Statistical analyses were conducted using Microsoft Excel and R software (version 4.0). The data set was assessed for departure from normality using the Shapiro Wilk test. As all the dependent variables were non-parametric, appropriate statistical analyses were used, including Pearson’s chi-squared goodness of fit test (followed by binary post-hoc tests for significant outcomes), Mann–Whitney U tests and Fisher’s exact test. Odds ratios (ORs) of significant Fisher’s *p* values are reported. Model significance was set at 0.05, and all tests were two tailed. Continuous data are reported as mean and standard deviation (SD) as well as median and interquartile range (IQR) and categorical data as counts and percentages.

The prevalence of COVID-19-related delusional content was calculated as a percentage of the total sample size, with 95% confidence intervals (CIs). Data are presented in tables, figures or described in the text.

#### Definitions used in the study

- A COVID-19-related delusion will be defined as any delusion in which COVID/COVID-19/corona/coronavirus is mentioned within the content of the delusion.
- Diagnosis: The discharge diagnosis was noted from the discharge summary. Patients were interviewed on admission by trainee psychiatrists and medical officers in psychiatry, with the final diagnosis being reviewed by the supervising psychiatrist according to DSM-5 criteria as per South African norms of practice.
- Substance use disorder: The substance use disorder diagnosis was noted from the discharge summary. The diagnosis was determined by the treating psychiatrist according to DSM-5 criteria as per South African norms of practice.
- Delusions were classified as persecutory, grandiose, religious, somatic, guilt, love, bizarre and referential.

#### The discharge diagnoses were simplified to fall into 1 of 6 possible overarching diagnoses

- Bipolar disorders (BD) (this included bipolar 1 disorder and bipolar 2 disorder).
- Psychotic and/or mood disorders because of another medical condition (AMC) (with delusions).
- Major depressive disorder with psychotic features (MDD).
- Schizoaffective disorders (SAD) (both depressive and bipolar subtypes).
- Schizophrenia and related disorders (SCZ and related) (schizophrenia, schizophreniform; brief psychotic disorder).
- Substance-induced psychotic and/or mood disorders.

### Ethical considerations

Permission to conduct the study was obtained from the Chief Executive Officer and the Head of Department of Psychiatry at CHBAH, the Deputy Registrar and Head of Department of Psychiatry at the University of Witwatersrand (WITS). Ethical approval was obtained by the WITS Human Research Ethics Committee (Medical) – clearance number M210621.

## Results

There were 791 patients admitted to the psychiatry unit during the period from 01 April 2020 until 30 September 2020 (6 months). A total of 193 did not meet inclusion criteria, 40 were excluded as they were under 18 years of age and 153 had no delusions at presentation. This left 598 files for review. Of these, 68 were excluded because of not having a psychiatric diagnosis and were transferred or discharged after an initial assessment. Of the remaining 530 eligible files, 451 files were traced and retrospectively analysed (79 were not found and/or untraceable).

### Sociodemographic characteristics

The sociodemographic and clinical characteristics of the study population are presented in Table 1. Most of the patients were male, single, had not completed school and were unemployed. A majority had a history of previous psychiatric diagnoses and were using substances. The mean age of the patients was 33.4 (SD = 10.9) years, with a range of 18 to 84 years old.

**TABLE 1:** Socio-demographic and clinical characteristics of acute admissions (*N* = 451).

Variable	*n*	%	Median	IQR
**Age**				
-	-	-	31	15
**Gender**				
Female	169	37.6	-	-
Male	282	62.4	-	-
**Highest level of education**				
Below grade 12	297	65.9	-	-
Matric and above	154	34.1	-	-
**Marital status**				
In a relationship	76	16.9	-	-
Single	375	83.1	-	-
**Employment status**				
Employed	31	6.9	-	-
Unemployed	420	93.1	-	-
**Past psychiatric history**				
Index	184	40.8	-	-
Previous episodes (readmission)	267	59.2	-	-
**Use of substances**				
Yes	258	57.2	-	-
No	193	42.8	-	-
**Discharge diagnosis**				
Bipolar disorders – with psychotic features	108	23.9	-	-
Psychosis and or mood disorder because of another medical condition	50	11.1	-	-
Major depressive disorder with psychotic features	10	2.2	-	-
Schizoaffective disorders	46	10.2	-	-
Schizophrenia	114	25.3	-	-
Substance-induced psychotic and mood disorders (with delusions)	123	27.3	-	-

IQR, interquartile range.

### Prevalence of COVID-19 related delusions

The prevalence of COVID-19-related delusional content among the patient sample was 25.5% (95% CI: 0.22-0.30; *n = 115).*

### Classification of COVID-19 related delusions

**FIGURE 1:**
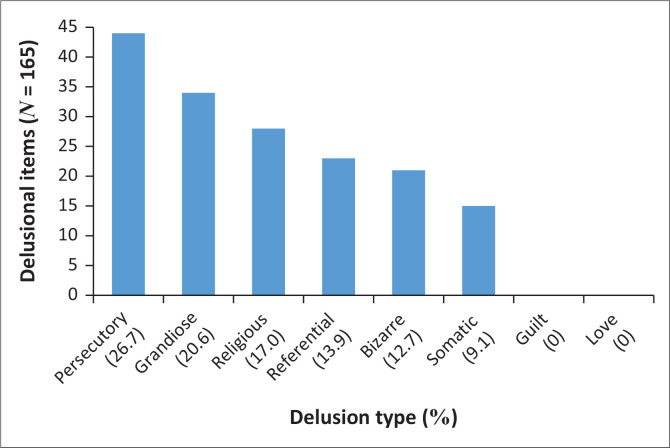
Classification of delusions in patients who presented with delusional content related to COVID-19.

Within the cohort of 115 patients experiencing COVID-19-related delusions, around one third (35/115) exhibited multiple delusions, spanning different delusional categories, resulting in a total of 165 delusional items. The most common type was persecutory, which represented 26.7% of the delusions, 20.6% were grandiose, 17.0% were religious, 13.9% were referential, 12.7% were bizarre and 9.1% were somatic in nature (Figure 1). Anecdotal delusions collected by the principal investigator include instances where patients believed they had contracted the virus through witchcraft or had been given COVID-19 intentionally (persecutory), possessed a unique cure for COVID-19 (grandiose delusion), interpreted COVID-19 as a divine message (religious delusion), perceived personal relevance in news coverage of the pandemic (referential delusion), attributed infection to exposure to cleaning products (bizarre delusion) and felt infested with COVID-19 particles or parasites (somatic delusion).

### Associations between COVID-19 delusional content and patient characteristics

The association between COVID-19-related delusional content and the patient characteristics are presented in Table 2.

**TABLE 2:** The relationship between demographic and clinical variables and COVID-19 related delusion content.

Variables	COVID-19-related delusion								*p*
	Presence (*N* = 115)				Absence (*N* = 336)				
	*n*	%	Median	IQR	*n*	%	Median	IQR	
**Age**									
Median in years [IQR]	-	-	31	15.5	-	-	32	15.0	-
**Sex**									0.885
Female	43	37.4	-	-	126	37.5	-	-	
Male	72	62.6	-	-	210	62.5	-	-	
**Highest level of education**									**0.000338**
Below grade 12	60	52.2	-	-	237	70.5	-	-	
Matric and above	55	47.8	-	-	99	29.5	-	-	
**Marital status**									0.492
In a relationship	17	14.8	-	-	59	17.6	-	-	
Single	98	85.2	-	-	277	82.4	-	-	
**Employment status**									0.080
Employed	12	10.4	-	-	19	5.7	-	-	
Unemployed	103	89.6	-	-	317	94.3	-	-	
**Past psychiatric history**									0.081
Index (first episode)	39	33.9	-	-	145	43.2	-	-	
Previous episodes (readmission)	76	66.1	-	-	191	36.8	-	-	
**Use of substances**									0.543
Yes	63	54.8	-	-	195	58.0	-	-	
No	52	45.2	-	-	141	42.0	-	-	

Note: Count and percentage data are shown. Statistics = Fisher’s exact test; significant outcomes are shown in bold. *U* or *W* = 19156, *P* = 0.89, *Z* = −0.13553.

IQR, interquartile range.

Age did not differ significantly between the COVID-19 related delusional and non-COVID-19-related delusional groups of patients (Mann-Whitney *U = W* = 19156; *p* = 0.89). In addition, gender, employment and relationship status did not differ significantly in their association with and without COVID-19-related delusion content (Table 2).

The only sociodemographic factor that was statistically more likely to be present among those with COVID-19-related delusions was education level. The OR of presenting with COVID-19-related delusional content was 2.19 for patients with a senior certificate compared to those with below grade 12 education (OR 2.19, 95% CI: [1.4-3.4]).

### Association of COVID-19 related delusions and clinical characteristics

Patients with index and previous psychiatric diagnoses had similar rates of COVID-19-related delusion content. The presence or absence of substance use was not found to have a statistically significant association with COVID-19-related delusional presentation.

### The relationship between COVID-19 related delusional content and the patient’s discharge diagnosis

The diagnoses found among the patients with COVID-19-related delusional content and those without are found in Table 3.

**TABLE 3:** Discharge diagnoses in patients with and without COVID-19 related delusional content.

Discharge diagnosis	COVID-19-related delusion				*p*-value
	Presence (*N* = 115)		Absence (*N* = 336)		
	*n*	%	*n*	%	
SCZ and related	35	30.4	79	23.5	0.431
BD	31	27.0	77	22.6	0.569
Substance-induced psychosis	27	23.5	96	28.6	0.095
SAD	14	12.2	32	9.5	0.994
Because of AMC	7	6.1	43	12.8	0.039
MDD	1	0.9	9	2.7	0.231

Note: Count and percentage data are shown. Statistics, chi-squared tests per variable; significant outcomes are shown in bold. Statistics: χ^2^ = 49.92, *df* = 5, *p* < 0.001; χ^2^ = 98.20, *df* = 5, *p* < 0.001.

SCZ and related, schizophrenia and related disorders; AMC, another medical condition; SAD, schizoaffective disorders; MDD, major depressive disorder; BD, bipolar disorders; *df*, degree of freedom.

Patients with COVID-19-related delusion content differed significantly in their discharge diagnosis (χ^2^ = 49.92, *df* = 5, *p* < 0.001). The most frequent diagnoses were schizophrenia and related disorders (30.4%), bipolar disorders (27%), substance-induced psychotic and/or mood disorders (23.5%). Followed by schizoaffective disorders (12.2%) and then psychotic disorders because of another medical condition (6.1%) and major depressive disorder in one patient (0.9%) (Table 3) (binary post-hoc tests).

Patients without COVID-19-related delusional content also differed significantly in their discharge diagnosis (χ^2^ = 98.20, *df* = 5, *p* < 0.001). The discharge diagnoses in the patients without COVID-19-related delusion content included substance-induced psychotic and/or mood disorders (28.6%), schizophrenia and related disorders (23.5%) and bipolar disorders (22.6%) followed by psychotic disorders because of another medical condition (12.8%) and major depressive disorder (2.7%) (Table 3) (binary post hoc tests).

The prevalence of each diagnosis was compared using a Fisher’s exact test. In the group of patients with COVID-19 delusional content; there was a statistically significant association between a diagnosis of psychotic disorder because of another medical condition and schizophrenia and related disorders (*p* = 0.032). The odds of COVID-19-related delusional content was 2.72 times greater in patients with schizophrenia and related disorders than those with psychotic disorder because of another medical condition (OR 2.72 95% CI [1.11-6.6]).

In order to further compare diagnoses, similar conditions were grouped together and compared to psychotic disorders because of AMC. However, none of the specific diagnostic categories showed statistical significance.

## Discussion

### Key findings

#### Prevalence of COVID-19 related delusional content

From the data, it is evident that the impact of the early months of the COVID-19 pandemic was so extreme that it influenced the content of delusions. This supports the hypothesis regarding the environmental (socio-cultural) impact on delusional content.^11^ A quarter of patients admitted during this study period at CHBAH had delusional content related to COVID-19. The rapid and widespread dissemination of COVID-19-related news is a potential explanation for the significant prevalence of COVID-19 delusional content found in this group of patients with psychosis. Consumption of traditional social media markedly increased at the start of the COVID-19 pandemic.^32^ Additionally, negative affect has been described in people with regular viewing of pandemic-related social media and has been showed to increase the risk for psychopathology in those with existing vulnerabilities.^33,37^

This study evaluates a period of 6 months from the beginning of the pandemic. It would be interesting to see whether there was a difference in the prevalence of delusional content across different time periods as was studied in a Spanish Observational study, which evaluated the prevalence of COVID-19-related content in a tertiary hospital in Spain. They found a decrease in prevalence from 38.5% to 13.7% over the first 3 months; however, their sample size of 19 patients makes it difficult to generalise their results.^37^ The researchers hypothesised that the initial surge could be attributed to the pandemic’s abrupt onset, while later admissions were triggered by the secondary social consequences of COVID-19, like social isolation and economic impact. The higher proportion of patients presenting with COVID-19-related delusional content in the present study may be explained by the prolonged lockdown conditions in South Africa, which more closely resemble the Spanish study’s earlier assessment period and the more severe socio-economic impact in the developing country’s (South African) setting. However, investigating the indirect effects of COVID-19 beyond delusional content was beyond the scope of this study.

A description of the classifications of delusions among the COVID-19 patients containing delusional sample was done and showed that persecutory delusions were the most frequent. The prevalence rate was similar to the cumulative rates reported across the most represented diagnoses.^38^ Persecutory delusions that describe a perceived threat of harm or death often of personal significance have been speculated to be associated with negative affective states and the experience of emotional distress.^39^ Their prevalence is thus expected in the context of an unexpected, potentially life-threatening pandemic. This finding could be because of messages from authorities emphasising the danger of spreading or catching the virus from others and the consequences thereof. Fear and mistrust may additionally have resulted from misinformation describing the potential threat of vaccinations.^26,40^ The prevalence of grandiose and religious delusions in the context of the pandemic are not necessarily contradictory as theories on thought formation but further hypothesise that delusions may exist as an irrational attempt to make meaning or provide purpose in the context of anomalous and anxiety provoking experiences. These delusions may thus represent ‘delusions as a defence’ and may represent attempts to feel safe, in control and alleviate the anxiety experience.^40^ Yet, the exact reasons as to why people present with the certain delusional themes remain speculative.^19^ This finding aligns with the cognitive model of delusion formation that highlights the significance of negative emotional states in the context of belief appraisal and the assignment of salience.^39,41^

#### The relationship between socio-demographic features and COVID-19 related delusional content

Interestingly the only socio-demographic factor significantly associated with the presence of COVID-19 delusions was a higher level of education. One can infer that individuals with a higher education level may have a potential for greater exposure to COVID-19-related information and better understanding of the issues surrounding the pandemic. A study assessing the impact of the COVID-19 pandemic on individuals with severe mental illness in India suggests that individuals from lower socioeconomic backgrounds, and education levels have lower awareness of COVID-19 because of factors such as limited access to the Internet, media and healthcare, as well as increased financial burden. This finding aligns with previous research by Wolf et al. and Zhong et al.^34^ On the other hand, reports also indicate that individuals with poor economic status, lower education levels and unemployment are at higher risk of developing mental disorders during the pandemic.^32^ This suggests socioeconomic factors not only influence awareness of COVID-19 but also contribute to mental health challenges during this time. Both sets of findings underscore the importance of considering socioeconomic disparities in addressing both public health awareness and mental health support during the pandemic. In the present study population, the potential moderating and protective effects of increased access to employment opportunities and improved socio-economic status among those with higher education levels may have been offset by the pandemic’s overarching negative impact on employment rates and financial security. This is evidenced by the 96.3% unemployment rate in the sample. Individual factors such as employment and relationship status may not have been sufficiently varied within the studied population to demonstrate significant associations.

#### The relationship between COVID-19 related delusional content and the patient’s diagnosis

A significant finding was that patients with mood and/or psychotic disorders because of another medical condition were significantly less likely than those with schizophrenia and related disorders to present with COVID-19-related delusions. This may have occurred because of specific symptoms in keeping with the particular medical diagnosis. In addition, the psychotic symptoms that are associated with underlying medical conditions are different, such as a higher incidence of visual hallucinations.^42^

Compared with the general population, individuals with schizophrenia may be particularly susceptible to the anxiety and distress surrounding the COVID-19 pandemic. Dopamine release dysfunction in underlying neurobiological pathways responsible for prediction error signalling and salience processing are theorised to be responsible for the risk of delusion formation and maintenance in schizophrenia. Chance environmental events that would ordinarily be disregarded as irrelevant are misperceived and interpreted as possessing specific significance, thereby establishing an emotional framework for a delusional interpretation of their meaning.^41^

Inadequate mental health care and non-adherence because of interruptions in continuity of care increased the risk of relapse among individuals with severe mental illness. Periods of relapse may be characterised by cognitive errors in reasoning, attention and cognitive biases of ‘jumping to conclusions’, inability to shift perspective and inflexibility to counter argument that increase the risk of delusion formation in the context of threat-based conspiracy theories and the anxiety provoking rhetoric that surrounded the COVID-19 pandemic on social media.^12,43^

## Limitations

Data were collected from the files of patients admitted at CHBAH who were acutely psychotic presenting during the pandemic. A limitation of this study is the absence of available data concerning the delusional content of patients in alternative settings with COVID-19-related delusional content. This lack of comparative data restricts the ability to fully contextualise and analyse the results of this specific population group. The sub-groups, such as employed patients or patients with a specific diagnosis, were relatively small, which impacted the elucidation of statistically significant results within these subgroups.

Data were obtained retrospectively and is consequently dependent on the quality of the records. Information regarding the participants COVID-19 status is not available as asymptomatic patients were not routinely tested on admission. The course of delusions and the effect of treatment were not determined.

## Conclusion

It is evident that the COVID-19 pandemic had a major impact on mental health and had a direct influence on delusional content in patients presenting to an acute psychiatric unit in South Africa. Such findings underscore the influence of socio-cultural factors on the formation of delusions and support the notion of delusional content’s plasticity.
